# Assessment of Lead and Mercury Exposure Levels in the General Population of Korea Using Integrated National Biomonitoring Data

**DOI:** 10.3390/ijerph18136932

**Published:** 2021-06-28

**Authors:** Jeong-Wook Seo, Young-Seoub Hong, Byoung-Gwon Kim

**Affiliations:** 1 Environmental Health Center, Dong-A University, Busan 49201, Korea; jw.selab@gmail.com (J.-W.S.); yshong@dau.ac.kr (Y.-S.H.); 2 Department of Preventive Medicin, Dong-A University, Busan 49201, Korea

**Keywords:** biomonitoring, data integration, general population, lead, mercury

## Abstract

In Korea, the estimated values of blood lead (Pb) and mercury (Hg) levels differ between two national-level biomonitors, namely the Korean National Environmental Health Survey and the Korea National Health and Nutrition Examination Survey. The present study used integrated data from these surveys to estimate the representative values of the change in concentration and recent distribution characteristics. The yearly trend of age-standardized exposure levels in regular adults was identified, and the geometric mean (GM) adjusted according to demographic characteristics was presented. Age-standardized GM for blood Pb and Hg in the integrated data was 2.06 and 3.64 μg/L in 2008, respectively, which decreased to 1.55 and 2.92 μg/L, respectively, by 2017. Adjusted GMs from most recently conducted surveys (2015–2017) were 1.61 and 2.98 μg/L for blood Pb and Hg, respectively. In particular, the adjusted percentage of blood Hg exceeding the reference value of 5 μg/L was 20.79%. While the blood Pb and Hg exposure levels are decreasing in Korea, the levels remain high relative to those in other countries. The Hg levels exceeded the reference value in many individuals. Therefore, continued biomonitoring must be conducted, and a reduction plan and exposure management are needed for harmful metals, including Hg.

## 1. Introduction

As the types of hazardous substances are becoming increasingly diverse and the frequency of exposure continues to increase, there is a growing need to assess their exposure levels and risks. In particular, the US Agency for Toxic Substances and Disease Registry (ATSDR) has developed the Priority List of Hazardous Substances (also known as Substance Priority List), based on the National Priorities List (NPL) of the US Environmental Protection Agency (EPA), which identifies hazardous substances requiring management, considering their frequency, toxicity, and potential for human exposure [1]. Of these, harmful metals, such as arsenic (As: rank 1), lead (Pb: rank 2), mercury (Hg: rank 3), and cadmium (Cd: rank 7), belong to the upper ranks.

After transitioning from media-based monitoring to receptor-based monitoring for exposure assessment of hazardous substances, including heavy metals, organizations such as the US Centers for Disease Control and Prevention (CDC) and the German Human Biomonitoring Commission (HBM Commission) have continued to conduct national-level biomonitoring in the general population. Such efforts are used to estimate the level of exposure to various hazardous substances and establish various exposure indices, such as the reference dose, reference concentration, and tolerable daily intake, which are used as evidence for risk assessment.

Since 2000, Korea has continued to conduct biomonitoring for exposure assessment of hazardous substances. Specifically, the Korean National Environmental Health Survey (KNEHS) conducted by the Korea National Institute of Environmental Research (KNIER) and the Korea National Health and Nutrition Examination Survey (KNHANES) conducted by the Korea Centers for Disease Control and Prevention (KCDC) are representative surveys targeting the general population. Such monitoring is designed to present representative values of Pb and Hg exposure levels.

The KNEHS and KNHANES strive to be probability sample surveys for estimating representative values for Korea. The survey results are used for various policies, research, public promotion, and educational data. However, differences exist in the estimated values of some indices, including heavy metals, between these datasets, which could result in slight differences in the results depending on the choice of data. The present study assessed the yearly trend and latest distribution of exposure levels based on the representative values for Korea derived from integrating these two datasets.

## 2. Materials and Methods

### 2.1. Data

#### 2.1.1. KNEHS

The KNEHS, which is an annual survey conducted in phases, with each phase covering three years, focuses on identifying the human exposure level to environmental hazards. For sampling, the enumeration districts in the Korean Population and Housing Census (PHC) were used as the sampling frame. The household was used as a sampling unit and a household member as the observational unit. The enumeration district in the PHC was considered the primary sampling unit (PSU), while the secondary sampling unit (SSU) comprised households and household members. The primary criterion for stratification of enumeration districts was based on administrative districts and coastal areas, while the secondary criterion for stratification was based on socioeconomic factors. The probability proportional to size systematic sampling was used to extract the enumeration districts from each layer. Households within the sampled districts were systematically sampled. The present study used adult data from phases 1 to 3 (2009–2017, n = 16,576) and included comparable demographic and lifestyle characteristics. All procedures related to the collection of biological samples and heavy metal analysis by KNEHS followed the standard guidelines of KNIER [2,3].

#### 2.1.2. KNHAENS

**Figure 1:**
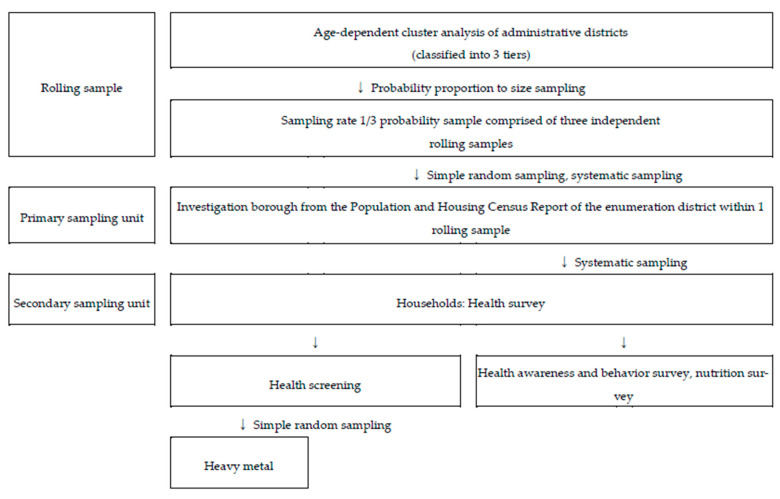
Korea National Health and Nutrition Examination Survey sampling design flow chart.

The KNHANES uses a rolling sampling design, similar to the US NHANES, for the assessment of exposure to hazardous substances and health impact. This survey was conducted in phases, with each phase covering three years, and consisted of annual rolling samples. It was designed to maintain the homogeneity of the characteristics of probability samples that represent the entire country. Accordingly, each rolling sample surveyed annually can be used to produce national statistics, and integrated survey results over three years could be used as the total sample survey data. Similar to the KNEHS, the sampling frame is the enumeration districts from the PHC, with households used as the sampling units and household members used as the observational units. The enumeration districts from the PHC was used for the PSU, and stratified cluster systematic sampling for selecting households was used for the SSU. For the selection of enumeration districts, the classifications of administrative regions and housing types were used as stratification characteristics. Moreover, simple random sampling was applied to some samples from the observational units of all samples for heavy metal analysis. The present study used adult data from 2005, 2008–2013, 2016, and 2017 (KNHANES III–VII, n = 18,869) when heavy metal analyses were performed, and it included comparable demographic and lifestyle characteristics (Figure 1). All procedures related to the collection of biological samples and heavy metal analysis followed the standard guidelines of the KNHANES [4].

#### 2.1.3. Data Integration

**Figure 2:**
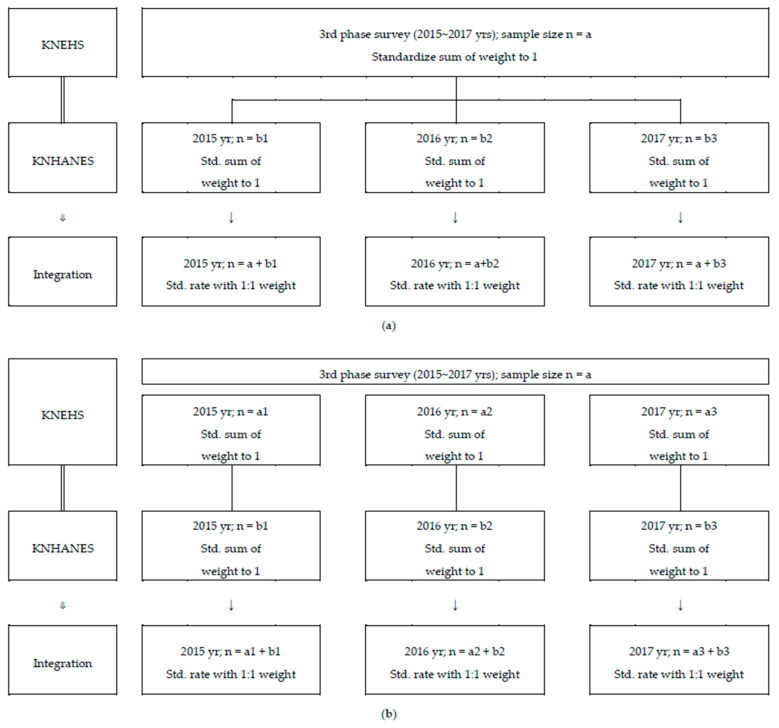
Example of KNEHS and KNHANES integrated data generation methods. (**a**) Method 1: Integrating phase data of KNEHS and yearly data of KNHANES to equal weights. (**b**) Method 2: Integrating yearly data of KNEHS and KNHANES to equal weights. KNEHS, Korean National Environmental Health Survey; KNHANES, Korea National Health and Nutrition Examination Survey; Std, standardized.

KNEHS and KNHANES surveys include sampling without replacement from a finite population, and they apply a weight to estimate the values that represent the general population. The weight applied to the two surveys reflects the population distribution of the PHC, and, thus, the weight was standardized, and data were integrated in a 1:1 ratio (Figure 2). Two methods were considered for data integration: KNEHS data integrated by phases (method 1) and years (method 2) based on annual rolling samples of KNHANES. With method 1, the estimated values for the phases of the KNEHS were applied equally to the corresponding three years. Consequently, yearly changes could not be reflected. The annual data of KNHANES consist of rolling samples, meaning they represent the entire population, whereas the annual data of KNEHS do not have such representativeness. Therefore, the integrated data from method 2 are limited in representing the entire population because they are from the probability sampling survey. There may be a problem with duplicate samples in the integrated data from the two surveys, but it has a low probability when considering sample size relative to the survey population.

### 2.2. Statistics Analysis

All statistics in the present study are weighted estimates, which were derived using the survey procedure of SAS version 9.4 (SAS Institute, Cary, NC). After the logarithmic conversion of blood Pb and Hg concentrations into a skewed distribution (skewness > 0), the SURVEYMEANS procedure was used to present the geometric mean (GM) and 95% confidence interval (CI) according to demographic and lifestyle characteristics. The SURVEYREG procedure was used to present the GM and 95% CI adjusted for demographic characteristics. To identify the yearly trends, age-standardized GM relative to the 2017 mid-year population in Korea was derived. All tests were conducted at a significance level of 5%.

## 3. Results

### 3.1. Combined Exposure Level of Blood Pb and Hg

**Figure 3:**
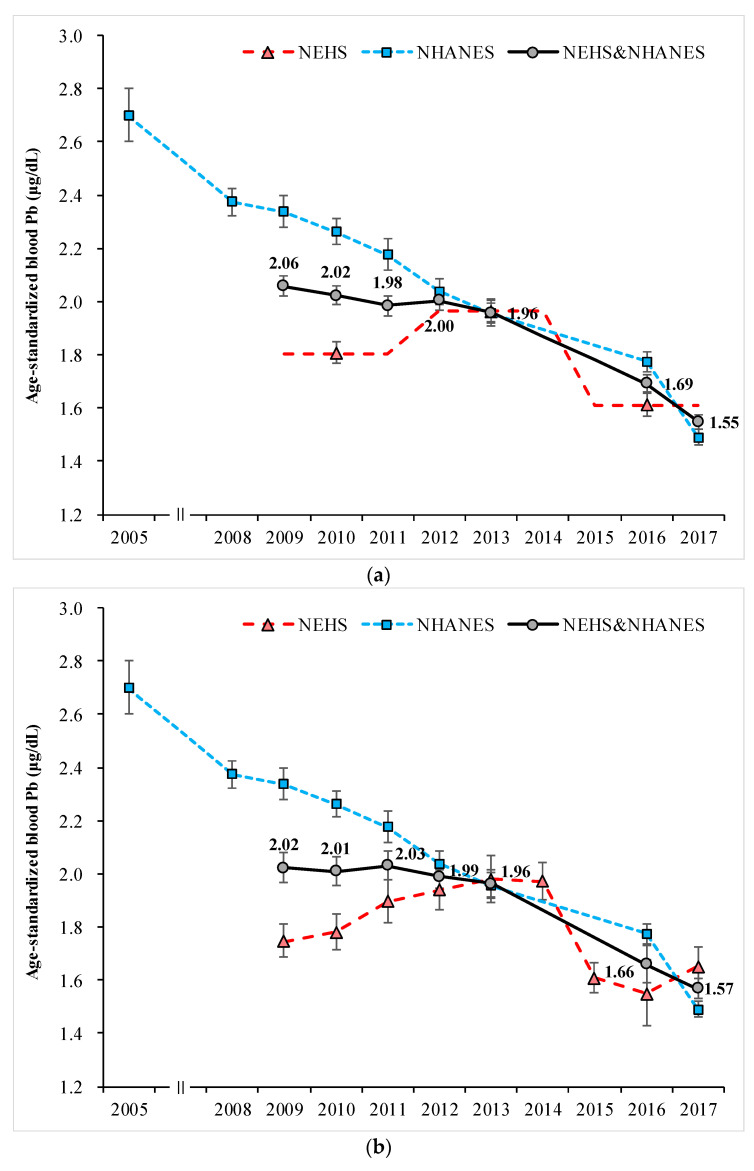

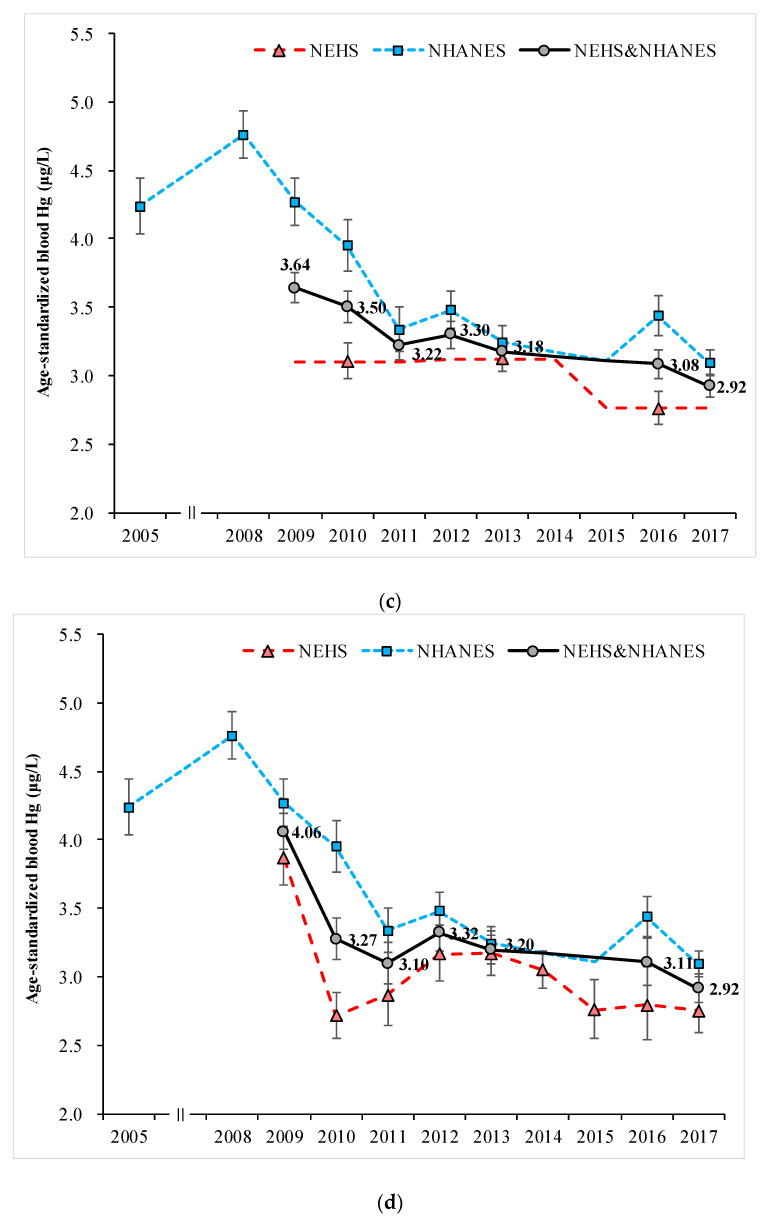
Geometric mean profiles of blood Pb and Hg concentrations. (**a**) Geometric mean profile of blood Pb concentration in integrated data using method 1; (**b**) geometric mean profile of blood Pb concentration in the integrated data by method 2; (**c**) geometric mean profile of blood Hg concentration in integrated data using method 1; (**d**) geometric mean profile of blood Hg concentration in integrated data using method 2. KNEHS, Korean National Environmental Health Survey; KNHANES, Korea National Health and Nutrition Examination Survey.

In the integrated data from each phase of the KNEHS and each year of the KNHANES (method 2), the weighted GM of blood Pb decreased from 2.01 in 2009 to 1.54 μg/dL in 2017 (1.98 in 2010, 1.95 in 2011, 1.97 in 2012, 1. in 2013, and 1.68 μg/dL in 2016). Similarly, blood Hg also decreased from 3.62 in 2009 to 2.92 μg/L in 2017 (3.48 in 2010, 3.20 in 2011, 3.28 μ in 2012, 3.19 in 2013, and 3.07 μg/L in 2016) (Table 1). The same trend was observed for age-standardized GM (Figure 3).

### 3.2. Blood Pb and Hg Concentrations According to Demographic Characteristics

Integrated data using a 1:1 ratio of data from the latest third phase of the KNEHS (2015–2017) and the seventh phase of the KNHANES (2016–2017) were used to assess blood Pb and Hg concentrations according to demographic characteristics. The integrated weighted GM of blood Pb adjusted for sex, age, smoking status, drinking status, household income, and education level was 1.61 μg/dL. Significantly higher concentrations were found among male and older populations compared to their counterparts. Statistically significant differences were also found according to smoking and drinking status. The adjusted integrated weighted GM of blood Hg was 2.98 μg/L, with estimates from KNHANES being slightly higher than that of KNEHS. The values were significantly higher in males than in females. Moreover, there was a statistically significant increasing trend with increasing age. However, people aged ≥ 70 yr showed a slight decrease compared to those aged 61–69 yr. In addition, the values were relatively higher among smokers and drinkers, while the values showed a statistically significant increase with higher household income and education level (Table 2).

### 3.3. Exceedance of Reference Values for Blood Pb and Hg According to Demographic Characteristics

In the latest integrated data, the exceedance of reference values was estimated as an indicator of exposure to high concentrations of Pb and Hg. The adjusted weighted exceedance rate for blood Pb (reference value of 5 μg/dL) was 0.77%. Males showed a notable exceedance rate as compared to females, and the difference was statistically significant. There was a general trend of higher distribution of high-dose exposure associated with older age, being a current smoker or drinker, lower household income, and lower education level. However, these differences were not statistically significant. The adjusted weighted exceedance rate for blood Hg (reference value of 5 μg/dL) was very high (20.79%). The exceedance rate among males was higher by more than two times, while a higher exceedance rate was associated with older age, being a current smoker or drinker, higher household income, and higher education level. There were statistically significant differences in the exceedance rates between these factors (Table 3).

## 4. Discussion

The present study used integrated data from the KNEHS and KNHANES, which represent the general adult population of Korea aged ≥ 20 yr, to estimate the characteristic distribution and latest trends in blood Pb and Hg concentrations in recent years.

With respect to the integrated data by method 1, the blood Pb concentration showed a decreasing trend. However, the levels remained high relative to the exposure levels among adults aged ≥ 20 yr reported in the US NHANES (2005–2006: 1.41, 2007–2008: 1.38, 2009–2010: 1.23, 2011–2012: 1.19, 2013–2014: 0.97, 2015–2016: 0.92 μg/dL) [5]. Compared to the findings in the Canadian Health Measures Survey (CHMS) conducted between 2007 and 2017 in the Canadian general population aged 6–79 yr, the exposure levels found in the present study were higher for the same time points, despite the difference in age (2007–2009: 1.5, 2009–2011: 1.3, 2012–2013: 1.1, 2014–2015: 0.95, 2016–2017: 0.93 μg/dL) [6,7]. Meanwhile, the German Environmental Survey (GerES) III conducted in 1998 in Germany on a German population aged 18–69 yr showed a blood Pb concentration of 3.16 μg/dL, which was slightly higher than that reported in the present study [8]. However, when compared to the adult exposure levels in 2006 (males: 2.10 and females: 1.35 μg/dL), reported in the study by Bierkens et al. [9], it is suspected that the levels are lower at the same time points.

In the present study, blood Pb levels were significantly associated with sex, age, smoking status, and amount of drinking. The findings in the 2015–2016 US NHANES (males: 0.92 and females: 0.73 μg/dL) [5] and various other studies [9,10,11] showed higher levels among males than females, which are consistent with the findings of the present study. With respect to age groups, the older age population showed higher values than the younger age population. A similar trend of linear increase in Pb concentration with increasing age was also found in the 2013–2014 US NHANES (males, 20–59 yr vs. ≥60 yr: 1.02 vs. 1.52 μg/dL; females, 20–59 yr vs. ≥60 yr: 0.71 vs. 1.28 μg/dL) [11], and 2016–2017 CHMS (20–39 yr: 0.78, 40–59 yr: 1.0, 60–79 yr: 1.4 μg/dL) [7]. In Korea, there was a linear increase up to the age of 60–69 yr, followed by a slight decrease at the age ≥ 70 yr. Current smokers showed higher Pb concentrations, which was consistent with the finding that smoking is a causal factor for increased blood Pb levels, as reported by previous studies [12,13,14,15]. Moreover, the findings were also consistent with studies reporting that drinking, among various lifestyle factors, was a factor that caused an increase in the Pb levels [13,16,17].

There was no study that exceeded the HBM I reference value of 15 μg/L for blood Pb levels proposed by the 2002 HBM commission. However, since a correlation between chronic low-dose exposure (≤10 μg/L) and health impact has been reported [18,19,20,21], it has been proposed that the existing reference value should be withdrawn [22] and management at a lower concentration would be needed. In fact, the CDC has proposed a reference value of 5 μg/dL [23]. In the present study, the integrated data by method 1 showed a generally decreasing trend in age-standardized annual exceedance rate with respect to the reference value of 5 μg/dL (2009: 2.40%, 2010: 1.85%, 2011: 1.93%, 2012: 2.45%, 2013: 1.58%, 2016: 0.96%, 2017: 0.60%). When the adjusted weighted 95th percentile values were compared, all the values in the present study (2009: 4.22, 2010: 4.09, 2011: 4.06, 2012: 3.95; 2013: 3.94, 2016: 3.34, 2017: 3.18 μg/dL) and US NHANES (2005–2006: 3.91, 2007–2008: 3.70, 2009–2010: 3.34, 2011–2012: 3.16, 2013–2014: 2.81, 2015–2016: 2.75 μg/dL) were below 5 μg/dL. However, the estimator was relatively higher in the present study [5]. The relative percentage of high-dose exposure was also higher.

In the integrated data by method 1, blood Hg concentration showed a relatively large decrease between 2008 and 2010, with a continuously decreasing trend. However, the levels were very high, as compared to those in participants aged ≥ 20 yr in US NHANES (2005–2006: 0.86, 2007–2008: 0.77, 2009–2010: 0.86, 2011–2012: 0.70, 2013–2014: 0.68, 2015–2016: 0.68 μg/L) [5], participants aged 6–79 yr in CHMS (2007–2009: 0.69, 2009–2011: 0.69, 2012–2013: 0.79, 2016–2017: 0.64 μg/L) (Haines 2017, Health Canada 2019), and participants aged 18–69 yr in GerES III (1990–1992: 0.50, 1998: 0.61 μg/L) [8].

In the present study, blood Hg levels were found to be correlated with sex, age, smoking status, drinking status, household income, and education level. In the 2015–2016 US NHANES (male 0.68, female: 0.68 μg/L) [5] and 2016–2017 CHMS (male 0.63, female 0.65 μg/L) [7], the levels were very low (approx. 0.6 μg/L) and were almost similar between the sexes. In our study, people aged 60–69 yr showed higher values than all other age groups. The concentration increased up to the age of 60–69 yr and decreased sharply at age ≥ 70 yr. In particular, the group aged ≥ 70 yr showed higher Hg concentration than the group aged 40–49 yr, while showing a large difference relative to the group aged 60–69 yr. In 2010–2011 US NHANES (20–29 yr: 0.66, 30–39 yr; 0.76, 40–49 yr: 0.89, 50–59 yr: 1.00, 60–69 yr: 1.13, and ≥70 yr: 0.87 μg/L), the same pattern of linear increase up to 60–69 yr followed by a decrease after ≥70 yr was found [24]. In 2016–2017 CHMS (20–39 yr: 0.55, 40–59 yr: 0.85, 60–79 yr: 0.83 μg/L), Hg concentration increased with increasing age up to ≤59 yr, but decreased again at ≥60 yr, showing a similar trend despite slight differences [7]. Similar to the present study, correlations of the concentration level with smoking [25,26,27,28] and drinking [27,29,30] status have been identified in some studies. Correlations with household income and education level have been similarly identified [27,31,32].

In the integrated data using method 1, the age-standardized annual exceedance rates for blood Hg showed a generally decreasing trend with respect to the HBM I reference value of 5 μg/L (2009: 29.96%, 2010: 28.66%, 2011: 24.17%, 2012: 24.82%; 2013: 22.78%, 2016: 22.69%, and 2017: 19.65%). The exceedance rate for HBM II reference value of 15 μg/L was also high (2009: 2.18%, 2010: 1.76%, 2011: 1.45%, 2012: 1.40%; 2013: 1.13%, 2016: 1.33%, 2017: 1.06%). Meanwhile, the adjusted weighted 95th percentile values were compared to identify the high-dose exposure level, which were ≥5 μg/L (2009: 11.28, 2010: 9.84, 2011: 9.67, 2012: 9.96; 2013: 9.46, 2016: 9.42, 2017: 8.59 μg/L), whereas all estimates, except some, were <5 μg/L in US NHANES (2005–2006: 4.64, 2007–2008: 4.64, 2009–2010: 5.13, 2011–2012: 4.40, 2013–2014: 4.36, 2015–2016: 4.25 μg/L) [5]. As the indicators of high-dose exposure, the exceedance rate and 95th percentile estimates were relatively higher in the present study.

Compared to the results from the studies conducted outside Korea, the blood Hg concentration in the Korean population showed a relatively large difference. This could be inferred based on various studies on the correlation between the amount of fish consumed and Hg concentration in the body [31,33,34,35,36,37,38,39] and a study reporting that Asians have higher fish consumption than other ethnicities [40]. In a report on the amount of seafood consumed by countries, Asian countries, including Korea, continue to rank high [41,42]. However, a series of studies represent outcomes based on relative comparisons within survey data, and thus, additional external validity is needed for generalization in comparisons between countries.

The present study proposed and used simple data integration methods to overcome differences in the estimated values of blood Pb and Hg from two different national-level biomonitoring systems in Korea. However, the datasets had different survey periods, phases, and time points for the target population and sampling design, and consequently, there were some limitations in integrating such data. Specifically, the proposed methods integrated data from KNEHS as a one-phase unit consisting of three years and data from the KNHANES as a one-year unit. Consequently, representativeness for each year can be viewed as a weakness. In addition, there is a difference between the estimates of the two datasets. This seems to be a limitation of estimating the representative value of 40 million people with a sample size of about 2000 per year. There are many factors influencing blood lead and mercury exposure, and an insufficient sample size for each level is likely to cause bias. Additional results for this are presented in Tables S1–S4. It is speculated that the effect of the non-sampling error before the system and protocol were stably established at the beginning of the surveys existed. Differences in estimates can be inferred from various causes, but not when clearly presented can also be pointed out as a limitation. Despite this, the integrated data that were generated presented unified representative values for blood Pb and Hg levels to eliminate confusion and promote consensus on inference. The findings in the present study are expected to be used as reference data in studies that compare the general population against vulnerable regions/populations and for comparisons with studies conducted outside Korea.

## 5. Conclusions

Blood Pb and Hg concentrations in the general adult population of Korea show a continuously decreasing trend. However, they remained relatively higher than those in other countries. The Hg concentration was particularly high. The findings of the present study are expected to be used as indicators that allow comparisons between countries. Moreover, the findings highlighted the need for continued biomonitoring of harmful metals and reiterated the need for reduction plans and management for high-dose exposure to harmful metals.

## Figures and Tables

**Table 1:** Compositional data.

Heavy Metal	Survey Data		Weighted GM (Stderr)										
	KNEHS Phase				1st			2nd			3rd		
	KNHANES Phase		3rd	4th		5th			6th			7th	
	Year		2005	2008	2009	2010	2011	2012	2013	2014	2015	2016	2017
**Blood Pb** **(μg/dL)**	Phase	KNEHS			1.768 (0.023)			1.940 (0.024)			1.603 (0.023)		
		KNHANES	2.611 (0.050)	2.306 (0.019)		2.119 (0.015)			1.935 (0.026)			1.617 (0.016)	
	Year	KNEHS			1.725 (0.040)	1.748 (0.037)	1.836 (0.044)	1.920 (0.042)	1.934 (0.043)	1.963 (0.040)	1.591 (0.031)	1.554 (0.062)	1.647 (0.042)
		KNHANES	2.611 (0.050)	2.320 (0.026)	2.293 (0.030)	2.215 (0.025)	2.142 (0.032)	2.005 (0.026)	1.935 (0.026)			1.764 (0.022)	1.482 (0.017)
		Integration by method 1			1.989 (0.032)	1.967 (0.028)	1.984 (0.030)	1.962 (0.026)	1.935 (0.025)			1.656 (0.036)	1.562 (0.022)
		Integration by method 2			2.014 (0.021)	1.979 (0.020)	1.947 (0.021)	1.972 (0.018)	1.938 (0.018)			1.682 (0.017)	1.541 (0.014)
**Blood Hg** **(μg/L)**	Phase	KNEHS			3.083 (0.066)			3.113 (0.050)			2.752 (0.066)		
		KNHANES	4.188 (0.100)	4.481 (0.060)		3.557 (0.048)			3.268(0.063)			3.253 (0.044)	
	Year	KNEHS			3.808 (0.105)	2.697 (0.086)	2.873 (0.124)	3.167 (0.106)	3.136 (0.089)	3.050 (0.074)	2.729 (0.113)	2.852 (0.138)	2.733 (0.084)
		KNHANES	4.188 (0.100)	4.729 (0.086)	4.247 (0.088)	3.916 (0.099)	3.324 (0.085)	3.456 (0.071)	3.268 (0.063)			3.427 (0.076)	3.088 (0.050)
		Integration by method 1			4.022 (0.070)	3.251 (0.076)	3.091 (0.079)	3.309 (0.069)	3.201 (0.055)			3.128 (0.088)	2.907 (0.052)
		Integration by method 2			3.619 (0.056)	3.475 (0.060)	3.202 (0.054)	3.280 (0.049)	3.189 (0.040)			3.073 (0.054)	2.916 (0.043)

GM (Stderr): geometric mean (standard error); KNEHS, Korean National Environmental Health Survey; KNHANES, Korea National Health and Nutrition Examination Survey.

**Table 2:** Blood lead and mercury concentration by characteristics using integrated data recent survey.

Factor	Level	3rd Phase KNEHS (‘15~‘17) and 7th Phase KNHANES (‘16~‘17) Integrated Data				
		n (%)	Weighted GM (95% CI)			
			Blood Pb (μg/dL)		Blood Hg (μg/L)	
Total	Crude	8618 (100.0)	1.61 (1.58–1.64)		2.99 (2.91–3.07)	
	Age standardized		1.62 (1.59–1.64)		3.01 (2.93–3.08)	
	Adjusted		1.61 (1.58–1.63)		2.98 (2.90–3.06)	
Survey	KNEHS	3787 (43.9)	1.60 (1.56–1.64)		2.81 (2.68–2.94)	
	KNHANES	4831 (56.1)	1.62 (1.59–1.64)		3.18 (3.09–3.27)	
	*p*-value		0.517		<.001	
Sex	Male	3797 (44.1)	1.87 (1.84–1.91)		3.60 (3.48–3.72)	
age standardized	Female	4821 (55.9)	1.40 (1.38–1.42)		2.51 (2.44–2.58)	
	*p*-value		<0.001		<0.001	
Sex	Male	3797 (44.1)	1.78 (1.75–1.82)		3.41 (3.28–3.55)	
	Female	4821 (55.9)	1.45 (1.42–1.48)		2.61 (2.53–2.70)	
	*p*-value		<0.001		<0.001	
Age(yr)	18–29	879 (10.2)	1.22 (1.17–1.27)	^a^	2.08 (1.95–2.23)	^a^
	30–39	1384 (16.1)	1.43 (1.39–1.47)	^b^	2.89 (2.77–3.02)	^b^
	40–49	1558 (18.1)	1.59 (1.56–1.63)	^c^	3.22 (3.09–3.35)	^cd^
	50–59	1852 (21.5)	1.89 (1.84–1.93)	^d^	3.45 (3.32–3.59)	^cd^
	60–69	1706 (19.8)	1.92 (1.87–1.98)	^d^	3.52 (3.34–3.70)	^d^
	≥70	1239 (14.4)	1.88 (1.81–1.96)	^d^	3.08 (2.87–3.31)	^bc^
	*p*-value		<0.001		<0.001	
Smoke	Currently	1527 (17.9)	1.82 (1.77–1.87)	^a^	3.16 (3.01–3.32)	^a^
	Former	1743 (20.4)	1.63 (1.59–1.68)	^b^	3.13 (2.99–3.27)	^a^
	Never	5276 (61.7)	1.53 (1.50–1.56)	^c^	2.88 (2.78–2.98)	^c^
	*p*-value		<0.001		0.003	
Drink	Currently	6081 (71.1)	1.64 (1.61–1.66)	^a^	3.05 (2.97–3.14)	^a^
	Former	1187 (13.9)	1.51 (1.46–1.55)	^b^	2.68 (2.57–2.80)	^b^
	Never	1282 (15.0)	1.53 (1.48–1.58)	^b^	2.85 (2.72–3.00)	^b^
	*p*-value		<.001		<.001	
House income	Low	1568 (18.2)	1.64 (1.59–1.70)		2.76 (2.62–2.91)	^a^
	Middle low	2715 (31.6)	1.63 (1.59–1.68)		2.86 (2.75–2.98)	^ab^
	Middle high	2265 (26.3)	1.59 (1.55–1.63)		3.02 (2.92–3.14)	^bc^
	High	2055 (23.9)	1.58 (1.54–1.62)		3.19 (3.06–3.34)	^c^
	*p*-value		0.135		<0.001	
Education level	Below elementary	2148 (25.7)	1.64 (1.59–1.69)		2.67 (2.54–2.82)	^a^
	Middle	1625 (19.4)	1.59 (1.54–1.64)		2.83 (2.70–2.97)	^a^
	High	2355 (28.1)	1.62 (1.58–1.65)		3.06 (2.95–3.17)	^b^
	Above college	2243 (26.8)	1.59 (1.55–1.62)		3.26 (3.14–3.38)	^c^
	*p*-value		0.160		<0.001	

All estimates, except crude and age standardized, were adjusted by sex, age, house income, smoking status, drinking status, education level.GM (95% CI): geometric means (95% confidence interval). ^abcd^: Bonferroni post hoc grouping; estimates with the same letter are not significantly different.

**Table 3:** Proportion above reference value of blood lead and mercury in integrated data recent survey.

Factor	Level	3rd Phase KNEHS (‘15~’17) and 7th Phase KNHANES (‘16~’17) Integrated Data				
		n (%)	Weighted % (95% CI)			
			Blood Pb ≥ 5 μg/dL		Blood Hg ≥ 5 μg/L	
Total	Crude	8618 (100.0)	0.75 (0.51–0.99)		20.99 (19.65–22.33)	
	Age standardized		0.76 (0.52–1.00)		21.18 (19.83–22.52)	
	Adjusted		0.77 (0.52–1.02)		20.79 (19.45–22.13)	
Survey	KNEHS	3787 (43.9)	0.98 (0.59–1.36)		19.11 (16.91–21.32)	
	KNHANES	4831 (56.1)	0.55 (0.27–0.83)		22.54 (20.82–24.26)	
	*p*-value		0.071		0.021	
Sex	Male	3797 (44.1)	1.30 (0.88–1.72)		30.84 (28.78–32.90)	
Age standardized	Female	4821 (55.9)	0.24 (0.09–0.39)		11.53 (10.22–12.85)	
	*p*-value		<0.001		<0.001	
Sex	Male	3797 (44.1)	1.06 (0.72–1.40)		28.09 (25.84–30.33)	
	Female	4821 (55.9)	0.49 (0.25–0.73)		13.64 (12.00–15.27)	
	*p*-value		<0.001		<0.001	
Age (yr)	18–29	879 (10.2)	0.75 (0.00–1.70)		5.72 (3.41–8.03)	^a^
	30–39	1384 (16.1)	0.37 (0.00–0.75)		17.37 (14.39–20.34)	^b^
	40–49	1558 (18.1)	0.42 (0.00–0.91)		23.63 (20.86–26.39)	^c^
	50–59	1852 (21.5)	1.21 (0.57–1.85)		26.81 (24.31–29.31)	^c^
	60–69	1706 (19.8)	0.83 (0.27–1.40)		29.76 (26.62–32.90)	^c^
	≥70	1239 (14.4)	1.26 (0.30–2.21)		24.24 (20.25–28.23)	^bc^
	*p*-value		0.226		<0.001	
Smoke	Currently	1527 (17.9)	1.72 (0.83–2.61)	^a^	24.35 (21.21–27.49)	^a^
	Former	1743 (20.4)	0.79 (0.19–1.40)	^ab^	23.17 (20.17–26.18)	^ab^
	Never	5276 (61.7)	0.44 (0.21–0.67)	^b^	18.80(17.05–20.54)	^c^
	*p*-value		0.023		0.004	
Drink	Currently	6081 (71.1)	0.81 (0.53–1.10)		21.99 (20.51–23.46)	^a^
	Former	1187 (13.9)	0.72 (0.24–1.21)		14.61 (12.11–17.10)	^b^
	Never	1282 (15.0)	0.55 (0.02–1.09)		19.57 (16.82–22.31)	^a^
	*p*-value		0.703		<0.001	
House income	Low	1568 (18.2)	1.06 (0.22–1.91)		16.30 (13.54–19.07)	^a^
	Middle low	2715 (31.6)	1.00 (0.39–1.62)		18.04 (15.95–20.13)	^ab^
	Middle high	2265 (26.3)	0.60 (0.23–0.98)		21.23 (19.09–23.38)	^bc^
	High	2055 (23.9)	0.55 (0.16–0.94)		25.55 (22.90–28.20)	^c^
	*p*-value		0.533		<0.001	
Education level	Below elementary	2148 (25.7)	1.06 (0.37–1.76)		16.87 (14.34–19.41)	^a^
	Middle	1625 (19.4)	1.11 (0.27–1.95)		18.27 (15.80–20.75)	^a^
	High	2355 (28.1)	0.52 (0.17–0.86)		22.29 (20.09–24.48)	^b^
	Above college	2243 (26.8)	0.58 (0.23–0.93)		23.84 (21.62–26.06)	^b^
	*p*-value		0.419		<0.001	

All estimates, except crude and age standardized, are adjusted by sex, age, house income, smoking status, drinking status, education level. GM (95% CI): geometric means (95% confidence interval).^abc^: Bonferroni post hoc grouping; estimates with the same letter are not significantly different.

## Data Availability

Restrictions apply to the availability of these data. KNEHS was obtained after requesting permission from the National Institute of Environmental Sciences at https://www.nier.go.kr/NIER/egovEngIndex.jsp, accessed on 26 June 2021. KNHANES was available from the Centers for Disease Control and Prevention at https://knhanes.kdca.go.kr/knhanes/sub03/sub03_02_05.do, accessed on 26 June 2021.

## Supplementary Materials

The following are available online at https://www.mdpi.com/article/10.3390/ijerph18136932/s1, Table S1: Concentration of blood lead in KNEHS, Table S2: Concentration of blood lead in KNHANES, Table S3: Concentration of blood mercury in KNEHS, Table S4: Concentration of blood mercury in KNHANES.
