# Supplementary material for: Assessment of Lead and Mercury Exposure Levels in the General Population of Korea Using Integrated National Biomonitoring Data
Source: Int J Environ Res Public Health. 2021 Jun 28;18(13):6932. doi: 10.3390/ijerph18136932 (PMC8297126; doi:10.3390/ijerph18136932)
Supplement: Supplementary file 1 [file ijerph-18-06932-s001.zip › ijerph-1231238-supplementary.pdf]

Supplementary Materials

Table S1. Concentration of blood lead in KNEHS

Factor	Level	Blood Pb of KNEHS (㎍/dL)								
		1st stage ('09–'11)			2nd stage ('12–'14)			3rd stage ('15–'17)		
		n (%)	Weight GM (95% CI)		n (%)	Weight GM (95% CI)		n (%)	Weight GM (95% CI)	
Total	Crude		1.77(1.72–1.81)			1.94(1.89–1.99)			1.60(1.56–1.64)	
	Age standardized	6311(38.1)	1.81(1.77–1.85)		6478(39.1)	1.96(1.92–2.01)		3787(22.8)	1.61(1.57–1.65)	
	Adjusted		1.77(1.73–1.81)			1.94(1.90–1.98)			1.60(1.56–1.64)	
Sex	Male	2928(46.4)	2.22(2.16–2.27)		2774(42.8)	2.32(2.26–2.38)		1648(43.5)	1.89(1.84–1.95)	
Age standardized	Female	3383(53.6)	1.49(1.45–1.53)		3704(57.2)	1.67(1.63–1.71)		2139(56.5)	1.37(1.34–1.41)	
	p-value		<0.001			<0.001			<0.001	
Sex	Male	2928(46.4)	2.02(1.96–2.09)		2774(42.8)	2.20(2.14–2.26)		1648(43.5)	1.78(1.73–1.84)	
	Female	3383(53.6)	1.55(1.51–1.60)		3704(57.2)	1.72(1.68–1.76)		2139(56.5)	1.44(1.39–1.49)	
	p-value		<0.001			<0.001			<0.001	
Age (yr)	18–29	747(11.8)	1.33(1.25–1.41)	a	537(8.3)	1.50(1.43–1.57)	a	281(7.4)	1.25(1.16–1.34)	a
	30–39	1184(18.8)	1.61(1.55–1.67)	b	1056(16.3)	1.75(1.69–1.82)	b	542(14.3)	1.44(1.38–1.50)	b
	40–49	1336(21.2)	1.81(1.76–1.87)	c	1226(18.9)	1.98(1.91–2.05)	c	621(16.4)	1.57(1.51–1.63)	c
	50–59	1496(23.7)	2.12(2.06–2.19)	d	1439(22.2)	2.26(2.18–2.33)	d	883(23.3)	1.88(1.81–1.96)	d
	60–69	1113(17.6)	2.18(2.10–2.27)	d	1335(20.6)	2.25(2.15–2.36)	d	935(24.7)	1.90(1.81–1.99)	d
	≥70	435(6.9)	2.05(1.91–2.20)	d	885(13.7)	2.24(2.13–2.35)	d	525(13.9)	1.80(1.68–1.93)	d
	p-value		<0.001			<0.001			<0.001	
Region	Seoul/Gyeonggido/Incheon/gangwondo	2504(39.7)	1.76(1.70–1.82)		–			–		
	Chungcheongdo	794(12.6)	1.78(1.68–1.89)		–			–		
	Jeollado	804(12.7)	1.86(1.76–1.97)		–			–		
	Gyeongsangdo	2042(32.4)	1.74(1.68–1.81)		–			–		
	Jejudo	167(2.6)	1.68(1.53–1.86)		–			–		
			0.317							
Smoke	Currently	1417(22.5)	2.01(1.95–2.08)	a	1161(17.9)	2.16(2.09–2.23)	a	604(15.9)	1.86(1.77–1.95)	a

	Former	1077(17.1)	1.83(1.75–1.92)	b	1058(16.3)	1.99(1.91–2.07)	b	760(20.1)	1.64(1.57–1.72)	b
	Never	3815(60.5)	1.66(1.61–1.71)	c	4259(65.7)	1.86(1.81–1.91)	c	2423(64.0)	1.52(1.47–1.56)	c
	p-value		<0.001			<0.001			<0.001	
Drink	Currently	3695(58.6)	1.88(1.83–1.93)	a	3824(59.0)	2.01(1.97–2.06)	a	2549(67.3)	1.63(1.58–1.67)	a
	Former	623(9.9)	1.64(1.56–1.72)	b	424(6.5)	1.80(1.68–1.93)	b	473(12.5)	1.51(1.44–1.59)	b
	Never	1986(31.5)	1.59(1.54–1.65)	b	2230(34.4)	1.82(1.76–1.88)	b	765(20.2)	1.54(1.47–1.61)	ab
	p-value		<0.001			<0.001			0.004	
House income	Low	1544(24.9)	1.76(1.68–1.84)		1827(28.2)	1.95(1.88–2.03)		715(18.9)	1.66(1.55–1.76)	
	Middle low	1728(27.8)	1.73(1.68–1.79)		2979(46.0)	1.95(1.90–1.99)		1524(40.2)	1.62(1.56–1.68)	
	Middle high	1317(21.2)	1.77(1.71–1.83)		1614(24.9)	1.91(1.84–1.98)		936(24.7)	1.58(1.51–1.65)	
	High	1624(26.1)	1.81(1.74–1.89)		58(0.9)	1.99(1.73–2.30)		612(16.2)	1.56(1.49–1.64)	
	p-value		0.420			0.712			0.421	
Education level	Below elementary	1217(19.3)	1.93(1.82–2.04)	a	1420(21.9)	2.09(2.00–2.19)	a	1273(33.6)	1.60(1.54–1.67)	
	Middle	905(14.3)	1.89(1.82–1.97)	a	874(13.5)	2.04(1.94–2.14)	a	1164(30.7)	1.56(1.50–1.62)	
	High	2042(32.4)	1.79(1.73–1.84)	a	1948(30.1)	1.97(1.91–2.03)	a	851(22.5)	1.64(1.57–1.71)	
	Above college	2147(34.0)	1.67(1.61–1.72)	b	2236(34.5)	1.86(1.80–1.92)	b	499(13.2)	1.63(1.56–1.70)	
	p-value		<0.001			0.002			0.184	

All estimates, except crude and age standardized, are adjusted by sex, age, house income, smoking status, drinking status, education level.

GM(95% CI): geometric means (95% Confidence Interval).

[abc]: Bonferroni post hoc grouping; estimates with the same letter are not significantly different.

Table S2. Concentration of blood lead in KNHANES.

Factor	Level	Blood Pb of KNHANES ($\mu g/dL$)							
		4th stage ('08–'09)		5th stage ('10–'12)		6th stage ('13)		7th stage ('16–'17)	
		n (%)	Weight GM (95% CI)	n (%)	Weight GM (95% CI)	n (%)	Weight GM (95% CI)	n (%)	Weight GM (95% CI)
Total	Crude		2.31(2.27–2.34)		2.12(2.09–2.15)		1.94(1.88–1.99)		1.62(1.59–1.65)
	Age standardized	3996(21.2)	2.35(2.31–2.39)	6050(32.1)	2.16(2.13–2.19)	1995(10.6)	1.98(1.93–2.03)	4831(25.6)	1.63(1.60–1.66)
	Adjusted		2.30(2.27–2.34)		2.11(2.09–2.14)		1.92(1.88–1.97)		1.61(1.59–1.64)

Sex	Male	1995(49.9)	2.78(2.73–2.84)		2976(49.2)	2.54(2.50–2.59)		996(49.9)	2.29(2.23–2.36)		2149(44.5)	1.86(1.82–1.90)
Age standardized	Female	2001(50.1)	2.00(1.95–2.04)		3074(50.8)	1.85(1.82–1.88)		999(50.1)	1.70(1.64–1.75)		2682(55.5)	1.43(1.40–1.46)
	p-value		<0.001			<0.001			<0.001			<0.001
Sex	Male	1995(49.9)	2.62(2.55–2.68)		2976(49.2)	2.39(2.34–2.44)		996(49.9)	2.17(2.10–2.25)		2149(44.5)	1.78(1.74–1.82)
	Female	2001(50.1)	2.04(1.98–2.09)		3074(50.8)	1.87(1.83–1.92)		999(50.1)	1.71(1.65–1.77)		2682(55.5)	1.47(1.43–1.50)
	p-value		<0.001			<0.001			<.001			<0.001
Age (yr)	18–29	789(19.7)	1.85(1.79–1.91)	a	1211(20.0)	1.64(1.59–1.68)	a	412(20.7)	1.51(1.44–1.58)	a	598(12.4)	1.21(1.16–1.25)
	30–39	804(20.1)	2.20(2.14–2.27)	b	1211(20.0)	1.98(1.93–2.03)	b	401(20.1)	1.82(1.74–1.91)	b	842(17.4)	1.44(1.40–1.49)
	40–49	800(20.0)	2.45(2.37–2.53)	c	1213(20.0)	2.23(2.17–2.29)	c	391(19.6)	2.05(1.96–2.13)	c	937(19.4)	1.64(1.59–1.69)
	50–59	791(19.8)	2.65(2.58–2.73)	d	1212(20.0)	2.49(2.42–2.55)	d	404(20.3)	2.22(2.13–2.30)	c	969(20.1)	1.88(1.83–1.94)
	60–69	492(12.3)	2.54(2.43–2.65)	cd	1018(16.8)	2.44(2.36–2.52)	e	343(17.2)	2.08(1.95–2.21)	c	771(16.0)	1.89(1.82–1.96)
	≥70	320(8.0)	2.44(2.30–2.59)	bcd	185(3.1)	2.29(2.14–2.46)	bcde	44(2.2)	2.24(2.00–2.50)	c	714(14.8)	1.92(1.84–2.00)
	p-value		<0.001			<0.001			<0.001			<0.001
Region	Seoul/Gyeonggido/Incheon/gangwondo	1884(47.1)	2.29(2.24–2.34)	a	3264(54.0)	2.07(2.03–2.10)	a	1112(55.7)	1.92(1.85–1.98)	a	2515(52.1)	1.58(1.55–1.62)
	Chungcheongdo	476(11.9)	2.35(2.23–2.48)	a	576(9.5)	2.27(2.18–2.36)	b	227(11.4)	1.87(1.73–2.04)	a	600(12.4)	1.68(1.59–1.76)
	Jeollado	505(12.6)	2.42(2.33–2.52)	a	585(9.7)	2.38(2.29–2.47)	b	176(8.8)	2.22(2.10–2.35)	b	505(10.5)	1.80(1.69–1.92)
	Gyeongsangdo	1052(26.3)	2.31(2.25–2.38)	a	1507(24.9)	2.09(2.03–2.14)	a	436(21.9)	1.86(1.76–1.96)	a	1111(23.0)	1.61(1.56–1.67)
	Jejudo	79(2.0)	1.76(1.56–1.99)	b	118(2.0)	1.81(1.56–2.10)	a	44(2.2)	2.09(1.76–2.49)	a	100(2.1)	1.40(1.27–1.55)
			<0.001			<0.001			<0.001			<0.001
Smoke	Currently	1034(26.0)	2.55(2.47–2.63)	a	1481(25.0)	2.32(2.26–2.39)	a	464(24.6)	2.10(2.01–2.19)	a	923(19.4)	1.78(1.73–1.83)
	Former	813(20.4)	2.35(2.28–2.42)	b	1203(20.3)	2.11(2.05–2.18)	b	344(18.2)	1.92(1.83–2.02)	b	983(20.7)	1.63(1.58–1.68)

	Never	2134(53.6)	2.18(2.12–2.24)	c	3232(54.6)	2.02(1.98–2.07)	b	1080(57.2)	1.86(1.80–1.92)	b	2853(59.9)	1.55(1.52–1.58)
	p-value		<0.001			<0.001			<0.001			<0.001
Drink	Currently	2974(74.6)	2.36(2.31–2.40)	a	4650(78.6)	2.16(2.13–2.20)	a	1470(77.7)	1.95(1.90–2.00)		3532(74.2)	1.64(1.62–1.67)
	Former	526(13.2)	2.17(2.09–2.24)	b	694(11.7)	1.99(1.92–2.07)	b	264(14.0)	1.81(1.70–1.92)		714(15.0)	1.50(1.44–1.55)
	Never	485(12.2)	2.15(2.06–2.24)	b	574(9.7)	1.91(1.82–1.99)	b	157(8.3)	1.93(1.82–2.05)		517(10.9)	1.53(1.47–1.59)
	p-value		<0.001			<0.001			0.570			<0.001
House income	Low	700(17.8)	2.35(2.26–2.44)		853(14.3)	2.18(2.11–2.26)		295(14.8)	1.97(1.87–2.08)		853(17.7)	1.64(1.59–1.70)
	Middle low	1007(25.7)	2.33(2.26–2.40)		1594(26.7)	2.10(2.05–2.16)		541(27.2)	1.88(1.80–1.96)		1191(24.7)	1.64(1.59–1.69)
	Middle high	1104(28.1)	2.27(2.22–2.33)		1739(29.1)	2.10(2.05–2.14)		554(27.9)	1.97(1.90–2.05)		1329(27.6)	1.61(1.56–1.65)
	High	1114(28.4)	2.29(2.23–2.35)		1789(29.9)	2.10(2.05–2.15)		597(30.0)	1.90(1.83–1.97)		1443(30.0)	1.59(1.55–1.64)
	p-value		0.462			0.284			0.164			0.415
Education level	Below elementary	874(22.0)	2.52(2.42–2.62)	a	977(16.5)	2.27(2.18–2.36)	a	300(15.9)	2.05(1.95–2.16)	a	875(19.1)	1.75(1.68–1.82)
	Middle	467(11.7)	2.44(2.35–2.53)	a	612(10.4)	2.26(2.18–2.35)	a	171(9.0)	2.08(1.92–2.24)	a	461(10.1)	1.71(1.64–1.78)
	High	1498(37.6)	2.28(2.23–2.34)	b	2237(37.9)	2.08(2.04–2.13)	b	746(39.5)	1.93(1.86–2.00)	ab	1504(32.8)	1.61(1.57–1.64)
	Above college	1140(28.7)	2.15(2.08–2.22)	c	2081(35.2)	2.02(1.97–2.06)	b	674(35.6)	1.83(1.75–1.90)	b	1744(38.0)	1.56(1.52–1.60)
	p-value		<0.001			<0.001			0.004			<0.001

All estimates, except crude and age standardized, are adjusted by sex, age, house income, smoking status, drinking status, education level.

GM (95% CI): geometric means (95% confidence interval).

[abc]: Bonferroni post hoc grouping; estimates with the same letter are not significantly different.

Table S3. Concentration of blood mercury in KNEHS.

Factor	Level	Blood Hg of KNEHS (㎍/L)								
		1st stage ('09–'11)			2nd stage ('12–'14)			3rd stage ('15–'17)		
		n (%)	Weight GM (95% CI)		n (%)	Weight GM (95% CI)		n (%)	Weight GM (95% CI)	
Total	Crude		3.08(2.96–3.21)			3.11(3.02–3.21)			2.75(2.63–2.88)	
	Age standardized	6311(38.1)	3.10(2.98–3.22)		6478(39.1)	3.12(3.03–3.21)		3787(22.8)	2.77(2.65–2.89)	
	Adjusted		3.10(2.98–3.23)			3.11(3.03–3.20)			2.75(2.63–2.87)	
Sex	Male	2928(46.4)	3.67(3.50–3.84)		2774(42.8)	3.70(3.57–3.83)		1648(43.5)	3.31(3.13–3.51)	
age standardized	Female	3383(53.6)	2.64(2.53–2.75)		3704(57.2)	2.65(2.56–2.73)		2139(56.5)	2.31(2.21–2.41)	
	p-value		<0.001			<0.001			<0.001	
Sex	Male	2928(46.4)	3.45(3.27–3.64)		2774(42.8)	3.48(3.35–3.62)		1648(43.5)	3.13(2.93–3.34)	
	Female	3383(53.6)	2.80(2.66–2.94)		3704(57.2)	2.79(2.68–2.90)		2139(56.5)	2.42(2.29–2.55)	
	p-value		<0.001			<0.001			<0.001	
Age(yr)	18–29	747(11.8)	2.25(2.08–2.44)	a	537(8.3)	2.28(2.15–2.42)	a	281(7.4)	1.85(1.66–2.06)	a
	30–39	1184(18.8)	3.11(2.92–3.31)	b	1056(16.3)	3.05(2.90–3.21)	bc	542(14.3)	2.76(2.57–2.96)	b
	40–49	1336(21.2)	3.38(3.18–3.60)	b	1226(18.9)	3.48(3.33–3.64)	d	621(16.4)	2.96(2.79–3.14)	bc
	50–59	1496(23.7)	3.84(3.62–4.08)	c	1439(22.2)	3.66(3.49–3.84)	d	883(23.3)	3.22(3.04–3.41)	c
	60–69	1113(17.6)	3.30(3.08–3.53)	b	1335(20.6)	3.43(3.23–3.65)	bd	935(24.7)	3.27(3.01–3.55)	c
	≥70	435(6.9)	2.94(2.65–3.28)	b	885(13.7)	2.89(2.68–3.11)	c	525(13.9)	2.76(2.44–3.12)	bc
	p-value		<0.001			<0.001			<0.001	
Region	Seoul/Gyeonggido/Incheon/Gangwon-do	2504(39.7)	2.93(2.76–3.11)	ab	–			–		
	Chungcheongdo	794(12.6)	2.41(2.11–2.76)	a	–			–		
	Jeollado	804(12.7)	2.91(2.67–3.17)	ab	–			–		
	Gyeongsangdo	2042(32.4)	3.85(3.56–4.16)	c	–			–		
	Jejudo	167(2.6)	4.04(3.06–5.32)	bc	–			–		
			<0.001							
Smoke	Currently	1417(22.5)	3.32(3.10–3.56)	a	1161(17.9)	3.38(3.21–3.56)	a	604(15.9)	2.90(2.66–3.16)	ab
	Former	1077(17.1)	3.26(3.06–3.48)	a	1058(16.3)	3.41(3.20–3.62)	a	760(20.1)	2.99(2.78–3.22)	a
	Never	3815(60.5)	2.97(2.83–3.11)	b	4259(65.7)	2.96(2.86–3.06)	b	2423(64.0)	2.63(2.49–2.78)	b
	p-value		0.004			<0.001			0.027	
Drink	Currently	3695(58.6)	3.27(3.13–3.42)	a	3824(59.0)	3.26(3.16–3.36)	a	2549(67.3)	2.81(2.67–2.95)	a

	Former	623(9.9)	2.68(2.49–2.89)	b	424(6.5)	2.69(2.46–2.94)	b	473(12.5)	2.50(2.33–2.68)	b
	Never	1986(31.5)	2.90(2.74–3.07)	b	2230(34.4)	2.89(2.78–3.01)	b	765(20.2)	2.66(2.50–2.84)	ab
	p-value		<0.001			<0.001			0.003	
House income	Low	1544(24.9)	3.10(2.88–3.34)		1827(28.2)	2.92(2.77–3.06)	a	715(18.9)	2.56(2.31–2.84)	
	Middle low	1728(27.8)	3.00(2.84–3.15)		2979(46.0)	3.08(2.98–3.18)	a	1524(40.2)	2.70(2.55–2.86)	
	Middle high	1317(21.2)	3.01(2.79–3.26)		1614(24.9)	3.34(3.20–3.49)	b	936(24.7)	2.78(2.63–2.95)	
	High	1624(26.1)	3.27(3.08–3.49)		58(0.9)	3.36(2.63–4.30)	ab	612(16.2)	2.89(2.66–3.14)	
	p-value		0.996			<0.001			0.240	
Education level	Below elementary	1217(19.3)	2.86(2.63–3.11)		1420(21.9)	2.98(2.77–3.20)		1273(33.6)	2.47(2.31–2.64)	a
	Middle	905(14.3)	3.13(2.91–3.36)		874(13.5)	3.00(2.83–3.19)		1164(30.7)	2.75(2.60–2.91)	b
	High	2042(32.4)	3.08(2.92–3.25)		1948(30.1)	3.13(3.01–3.25)		851(22.5)	2.91(2.73–3.11)	b
	Above college	2147(34.0)	3.21(3.03–3.39)		2236(34.5)	3.16(3.05–3.28)		499(13.2)	2.98(2.76–3.23)	b
	p-value	0.966			0.403				<0.001	

All estimates, except crude and age standardized, are adjusted by sex, age, house income, smoking status, drinking status, education level.

GM (95% CI): geometric means (95% confidence interval).

[abc]: Bonferroni post hoc grouping; estimates with the same letter are not significantly different.

Table S4. Concentration of blood mercury in KNHANES.

Factor	Level	Blood Hg of KNHANES (μg/L)							
		4th stage ('08–'09)		5th stage ('10–'12)		6th stage ('13)		7th stage ('16–'17)	
		n (%)	Weight GM (95% CI)	n (%)	Weight GM (95% CI)	n (%)	Weight GM (95% CI)	n (%)	Weight GM (95% CI)
Total	Crude		4.48(4.36–4.60)		3.56(3.46–3.65)		3.27(3.15–3.39)		3.25(3.17–3.34)
	Age standardized	3996(21.2)	4.48(4.37–4.61)	6050(32.1)	3.58(3.49–3.68)	1995(10.6)	3.30(3.15–3.45)	4831(25.6)	3.26(3.18–3.35)
	Adjusted		4.48(4.38–4.59)		3.56(3.47–3.65)		3.25(3.15–3.36)		3.25(3.16–3.34)
Sex	Male	1995(49.9)	5.43(5.25–5.62)	2976(49.2)	4.25(4.11–4.40)	996(49.9)	3.84(3.62–4.07)	2149(44.5)	3.91(3.78–4.04)

Age standardized	Female	2001(50.1)	3.73(3.62–3.84)		3074(50.8)	3.04(2.94–3.14)		999(50.1)	2.81(2.67–2.96)		2682(55.5)	2.73(2.64–2.82)	
	p-value		<0.001			<0.001			<0.001			<0.001	
Sex	Male	1995(49.9)	5.22(5.03–5.41)		2976(49.2)	4.02(3.87–4.17)		996(49.9)	3.59(3.41–3.77)		2149(44.5)	3.79(3.65–3.93)	
	Female	2001(50.1)	3.87(3.74–4.00)		3074(50.8)	3.16(3.05–3.27)		999(50.1)	2.96(2.82–3.10)		2682(55.5)	2.80(2.70–2.90)	
	p-value		<0.001			<0.001			<0.001			<0.001	
Age(yr)	18–29	789(19.7)	3.61(3.45–3.78)	a	1211(20.0)	2.73(2.61–2.86)	a	412(20.7)	2.35(2.19–2.52)	a	598(12.4)	2.43(2.28–2.59)	a
	30–39	804(20.1)	4.27(4.09–4.46)	b	1211(20.0)	3.43(3.29–3.58)	b	401(20.1)	2.95(2.78–3.13)	b	842(17.4)	3.12(2.98–3.27)	b
	40–49	800(20.0)	5.03(4.79–5.27)	cd	1213(20.0)	3.85(3.70–4.01)	c	391(19.6)	3.58(3.35–3.83)	c	937(19.4)	3.60(3.42–3.78)	c
	50–59	791(19.8)	5.22(5.00–5.45)	c	1212(20.0)	4.24(4.05–4.44)	d	404(20.3)	3.86(3.62–4.11)	c	969(20.1)	3.69(3.52–3.88)	c
	60–69	492(12.3)	4.65(4.37–4.95)	bd	1018(16.8)	3.90(3.66–4.14)	cd	343(17.2)	3.75(3.49–4.03)	c	771(16.0)	3.60(3.38–3.84)	c
	≥70	320(8.0)	4.36(3.99–4.76)	d	185(3.1)	3.59(3.16–4.07)	bcd	44(2.2)	3.44(2.67–4.43)	abc	714(14.8)	3.26(2.99–3.55)	bc
	p-value		<0.001			<0.001			<0.001			<0.001	
Region	Seoul/Gyeonggido/Incheon/gangwondo	1884(47.1)	4.22(4.09–4.36)	a	3264(54.0)	3.29(3.19–3.38)	a	1112(55.7)	2.96(2.83–3.10)	ab	2515(52.1)	3.02(2.92–3.11)	a
	Chungcheongdo	476(11.9)	3.63(3.38–3.90)	b	576(9.5)	3.00(2.78–3.25)	a	227(11.4)	2.77(2.59–2.95)	a	600(12.4)	3.07(2.82–3.34)	a
	Jeollado	505(12.6)	4.50(4.24–4.79)	a	585(9.7)	3.35(3.08–3.65)	a	176(8.8)	3.46(3.08–3.88)	bc	505(10.5)	3.31(3.08–3.56)	a
	Gyeongsangdo	1052(26.3)	5.43(5.14–5.73)	c	1507(24.9)	4.46(4.20–4.73)	b	436(21.9)	4.17(3.87–4.49)	c	1111(23.0)	3.90(3.65–4.17)	b
	Jejudo	79(2.0)	5.83(4.94–6.87)	c	118(2.0)	5.51(4.70–6.46)	b	44(2.2)	5.83(3.87–8.76)	c	100(2.1)	5.08(4.07–6.32)	b
			<0.001			<0.001			<0.001			<0.001	
Smoke	Currently	1034(26.0)	4.65(4.46–4.85)		1481(25.0)	3.88(3.70–4.07)	a	464(24.6)	3.44(3.21–3.69)	a	923(19.4)	3.42(3.24–3.60)	
	Former	813(20.4)	4.58(4.34–4.83)		1203(20.3)	3.62(3.45–3.79)	ab	344(18.2)	3.64(3.37–3.94)	a	983(20.7)	3.28(3.10–3.46)	
	Never	2134(53.6)	4.37(4.23–4.52)		3232(54.6)	3.39(3.28–3.51)	b	1080(57.2)	3.06(2.92–3.21)	b	2853(59.9)	3.18(3.07–3.29)	

	p-value		0.057			<0.001			0.001			0.076	
Drink	Currently	2974(74.6)	4.62(4.50–4.74)	a	4650(78.6)	3.64(3.54–3.74)	a	1470(77.7)	3.33(3.21–3.44)	a	3532(74.2)	3.33(3.24–3.43)	a
	Former	526(13.2)	3.96(3.77–4.15)	b	694(11.7)	3.10(2.92–3.30)	b	264(14.0)	2.94(2.73–3.18)	b	714(15.0)	2.86(2.71–3.02)	b
	Never	485(12.2)	4.26(3.98–4.55)	ab	574(9.7)	3.53(3.26–3.81)	a	157(8.3)	3.13(2.77–3.54)	ab	517(10.9)	3.14(2.90–3.39)	ab
	p–value		<0.001			<0.001			0.014			<0.001	
House income	Low	700(17.8)	4.13(3.92–4.36)	a	853(14.3)	3.30(3.09–3.52)	a	295(14.8)	2.99(2.74–3.27)	ab	853(17.7)	2.91(2.74–3.10)	a
	Middle low	1007(25.7)	4.28(4.12–4.44)	a	1594(26.7)	3.39(3.25–3.53)	a	541(27.2)	2.90(2.75–3.07)	a	1191(24.7)	3.09(2.95–3.23)	ab
	Middle high	1104(28.1)	4.45(4.27–4.64)	a	1739(29.1)	3.56(3.42–3.70)	a	554(27.9)	3.32(3.14–3.51)	b	1329(27.6)	3.28(3.14–3.42)	bc
	High	1114(28.4)	4.92(4.72–5.12)	b	1789(29.9)	3.92(3.76–4.08)	b	597(30.0)	3.67(3.48–3.87)	c	1443(30.0)	3.49(3.34–3.65)	c
	p-value		<0.001			<0.001			<0.001			<0.001	
Education level	Below elementary	874(22.0)	4.23(3.97–4.51)	a	977(16.5)	3.33(3.10–3.57)	a	300(15.9)	3.11(2.83–3.42)		875(19.1)	3.24(3.03–3.47)	ab
	Middle	467(11.7)	4.44(4.17–4.71)	ab	612(10.4)	3.64(3.43–3.86)	ab	171(9.0)	3.42(3.07–3.80)		461(10.1)	3.20(2.99–3.42)	ab
	High	1498(37.6)	4.41(4.28–4.55)	a	2237(37.9)	3.50(3.38–3.63)	a	746(39.5)	3.15(3.01–3.30)		1504(32.8)	3.14(3.01–3.27)	a
	Above college	1140(28.7)	4.78(4.59–4.98)	b	2081(35.2)	3.75(3.61–3.89)	b	674(35.6)	3.39(3.20–3.59)		1744(38.0)	3.36(3.24–3.49)	b
	p-value		0.002			0.002			0.076			0.052	

All estimates, except crude and age standardized, are adjusted by sex, age, house income, smoking status, drinking status, education level.

GM (95% CI): geometric means (95% confidence interval).

[abc]: Bonferroni post hoc grouping; estimates with the same letter are not significantly different.
